# Beak and feather disease virus (BFDV) prevalence, load and excretion in seven species of wild caught common Australian parrots

**DOI:** 10.1371/journal.pone.0235406

**Published:** 2020-07-01

**Authors:** Johanne M. Martens, Helena S. Stokes, Mathew L. Berg, Ken Walder, Shane R. Raidal, Michael J. L. Magrath, Andy T. D. Bennett

**Affiliations:** 1 Centre for Integrative Ecology, School of Life and Environmental Sciences, Deakin University, Waurn Ponds, Victoria, Australia; 2 Centre for Molecular and Medical Research, School of Medicine, Deakin University, Waurn Ponds, Victoria, Australia; 3 School of Animal and Veterinary Sciences, Faculty of Science, Charles Sturt University, Wagga Wagga, Australia; 4 Wildlife Conservation and Science, Zoos Victoria, Parkville, Victoria, Australia; Justus-Liebeig University Giessen, GERMANY

## Abstract

Pathogens pose a major risk to wild host populations, especially in the face of ongoing biodiversity declines. Beak and feather disease virus (BFDV) can affect most if not all members of one of the largest and most threatened bird orders world-wide, the Psittaciformes. Signs of disease can be severe and mortality rates high. Its broad host range makes it a risk to threatened species in particular, because infection can occur via spill-over from abundant hosts. Despite these risks, surveillance of BFDV in locally abundant wild host species has been lacking. We used qPCR and haemagglutination assays to investigate BFDV prevalence, load and shedding in seven abundant host species in the wild in south-east Australia: Crimson Rosellas (*Platycercus elegans*), Eastern Rosellas (*Platycercus eximius*), Galahs (*Eolophus roseicapillus*), Sulphur-crested Cockatoos (*Cacatua galerita*), Blue-winged Parrots (*Neophema chrysostoma*), Rainbow Lorikeets (*Trichoglossus moluccanus*) and Red-rumped Parrots (*Psephotus haematonotus*). We found BFDV infection in clinically normal birds in six of the seven species sampled. We focused our analysis on the four most commonly caught species, namely Crimson Rosellas (BFDV prevalence in blood samples: 41.8%), Sulphur-crested Cockatoos (20.0%), Blue-winged Parrots (11.8%) and Galahs (8.8%). Species, but not sex, was a significant predictor for BFDV prevalence and load. 56.1% of BFDV positive individuals were excreting BFDV antigen into their feathers, indicative of active viral replication with shedding. Being BFDV positive in blood samples predicted shedding in Crimson Rosellas. Our study confirms that BFDV is endemic in our study region, and can inform targeted disease management by providing comparative data on interspecies variation in virus prevalence, load and shedding.

## Introduction

Wildlife diseases pose major threats to biodiversity; data on the abundance and distribution of pathogens in natural populations are however often lacking, which impedes conservation efforts [1, 2]. Generalist pathogens with high mutation rates pose a particularly high risk for disease-induced extinction as they may be maintained in abundant reservoir hosts and thus tend not to be affected by population declines of a single host species [1, 3]. Consequently, host species that act as reservoirs for generalist pathogens can have a severe impact on co-occurring threatened host species, by maintaining infection and facilitating spill-over infections [4–6]. Understanding pathogen prevalence and shedding in abundant host species is therefore crucial when managing disease risk for threatened populations [4, 7].

Not all hosts are equally likely to be infected and to transmit pathogens, so pathogen prevalence and transmission rates can show high heterogeneity between host species [8, 9]. This heterogeneity can be caused by fluctuations in host resistance and tolerance to pathogens [6, 10], and by temporal and geographical factors influencing pathogen occurrence [11]. Prevalence and load can also be influenced by host sex, due to sex-specific immune responses [12]. Such host individual- and species-specific effects can influence the outcome of infections and can thus often be important for the management of wildlife disease [13].

Beak and feather disease virus (BFDV) is a globally distributed pathogen of one of the most threatened bird orders world-wide, the Psittaciformes (parrots, cockatoos, lorikeets) [5]. It has recently also been found in non-psittacine bird species, showing the capacity for spill-over infections to taxa outside the Psittaciformes [14–16]. BFDV is a single-stranded DNA circovirus with a circular genome of only about 2000 nucleotides [17], making it one of the smallest known viruses. It is particularly prone to mutations [18–20]. This facilitates flexible host-switching, enabling the virus to become a host generalist, and to maintain virulence [21–23]. BFDV prevalence and load can vary even between subspecies and hybrids within the same host species [24], possibly due to variation in host genetic diversity [25]. The influence of sex on BFDV prevalence and load has received little investigation, and existing data in wild birds are conflicting, either showing presence [24, 26] or absence [25, 27] of sex differences.

BFDV can cause mortality and potentially extinction [19, 20] and is therefore of global conservation concern [28]. It can be transmitted directly from bird to bird through feces, contaminated feather dust and crop secretions [29], as well as from mother to embryonated egg [30]. It is thought to be persistent in the host, as some host individuals can stay infected for several months [31]. Additionally, it appears to persist in the environment [32] and indirect transmission in co-occurring host species through contaminated nesting material has been suggested [26]. BFDV prevalence can be high, reaching 100% in some populations [33]. Such high prevalence is of concern particularly where reservoir host species co-occur with threatened species [33], as BFDV can be transmitted via spill-over from abundant hosts [4]. Presence of BFDV in small host populations of endangered psittacines has been documented in species such as the Orange-bellied Parrot (*Neophema chrysogaster*) [4] and Swift Parrot (*Lathamus discolor*) [34] in Australia, and in the Echo Parakeet (*Psittacula eques*) on Mauritius [19]. Mortality data from wild populations are however absent. BFDV causes one of the most widespread diseases of Psittaciformes, Psittacine Beak and Feather Disease (PBFD) [19], with feather loss and a deformed beak and claws being the main signs of disease [35]. Severity of clinical signs varies between host species [32, 36, 37]. Subclinically infected birds may act as BFDV reservoirs and potentially shed BFDV for extended periods, as they do not die from the disease [31, 38].

BFDV is thought to be endemic to Australia [39], and in 2005, it was declared a “key threatening process to biodiversity” by the Australian national government [40]. A paucity of data on BFDV prevalence and excretion rates from diseased and carrier birds in free-living populations remains a severe limitation on management strategies, and was identified as a key knowledge gap both in the Australian BFDV Threat Abatement Plan [40] and its review seven years later [41]. Despite this, there have been few studies of BFDV in wild birds, and they have tended to use small sample sizes on single host species [39] and/or specimens brought into veterinary care, which are likely to be a biased sample of diseased birds [16]. Only two studies have investigated species-specific BFDV prevalence in wild hosts which were not derived from vet clinics: one showing variation in prevalence between subspecies and hybrids within Crimson Rosellas due to host genetic variation [25] and one showing high BFDV prevalence, ranging from 56.7% to 100%, within the Cacatuidae family [39]. These studies however did not use a phylogenetically wide range of hosts, and did not determine whether infected birds also shed the virus, which would have implications for BFDV transmission dynamics. Most studies solely use PCR-based detection assays, as these are readily available and allow high sample throughput [28]. Only analyses with multiple BFDV detection methods (e.g. quantitative real-time PCR (qPCR) to investigate prevalence and load [42] and haemagglutination (HA) assays to determine active infection and excretion of viable antigen [43]) can distinguish between birds with active infection that shed the virus, and carrier birds that do not excrete viable antigen [44]. Data on both BFDV prevalence and shedding across a wide range of wild host species are needed to understand BFDV distribution and potential impacts in the wild [33].

In this study, we aimed to investigate the distribution of BFDV among locally abundant wild host species. We also aimed to reveal the role of host species and host sex in BFDV prevalence, load and shedding from wild-caught birds representing a wide phylogenetic host range. We expected interspecies variation in virus prevalence, load and shedding, as host responses have been shown to be species-specific: Crimson Rosellas are reported to show only mild signs of disease [32], whereas Sulphur-crested Cockatoos often show severe signs of BFDV infection [45]. We utilized multiple sample types (blood and cloacal swabs, feathers) and BFDV detection methods (qPCR, HA) to investigate the role of abundant species as BFDV carriers and shedders. We also tested the effect of BFDV infection on body condition and packed cell volume (PCV), which are both commonly used to estimate condition and health of hosts [46].

We tested seven species of parrots: Crimson Rosellas (*Platycercus elegans*, Psittacidae), Eastern Rosellas (*P*. *eximius*, Psittacidae), Galahs (*Eolophus roseicapillus*, Cacatuidae), Sulphur-crested Cockatoos (*Cacatua galerita*, Cacatuidae), Blue-winged Parrots (*Neophema chrysostoma*, Psittacidae), Red-rumped Parrots (*Psephotus hematonotus*, Psittacidae) and Rainbow Lorikeets (*Trichoglossus moluccanus*, Psittacidae). These species are common with overlapping distributions, and cover a broad phylogenetic range in the Psittaciformes order [47]. Signs of BFDV infection can vary considerably between these species: in Crimson Rosellas for example, which are thought to act as a BFDV reservoir [24], signs of infection with BFDV range from non-existent to mild [32], whereas Sulphur-crested Cockatoos often show severe signs when infected with BFDV, with abnormal beak growth and feather loss as the main visible signs, as well as tissue, organ and immune system damage [36, 45]. Blue-winged Parrots were included in this study because they may act as a potential reservoir host for the critically endangered Orange-bellied Parrot (*Neophema chrysogaster*). These two species are closely related and are known to co-occur in the same habitat, however Blue-winged Parrots are much more common [48]. Orange-bellied Parrots have been severely impacted by BFDV infection in the past, with infection suggested to originate from a spill-over event from a reservoir, probably one or several common parrot species [4]. To our knowledge, there are no data on signs of BFDV infection in Blue-winged Parrots. We focused our statistical analysis on the four most commonly trapped species, hereafter referred to as ‘focal species’, namely Crimson Rosellas, Galahs, Sulphur-crested Cockatoos and Blue-winged Parrots.

## Materials and methods

### Sample collection

This study was carried out in strict accordance with the recommendations in the Australian Code of Practice for the Care and Use of Animals for Scientific Purposes. The protocol was approved by the Animal Ethics Committee—Wildlife of Deakin University (AECW-B) (permit B31-2015). We conducted our work under Australian Bird and Bat Banding authority 2319, and complied with the laws of Victoria (research permit 10007969). All efforts were made to minimize suffering. We trapped six of the seven host species (Crimson Rosellas, Eastern Rosellas, Galahs, Sulphur-crested Cockatoos, Red-rumped Parrots, Rainbow Lorikeets) in baited walk-in cage traps at our field sites in Bellbrae (S38°19’ E144°11’), Meredith (S37°51’ E144°06’) and Steiglitz (37°52’ E144°18’) in the state of Victoria, in south-east Australia, between April 2017 and August 2018. We trapped the seventh host species, Blue-winged Parrots, in mist nets in Bellbrae. All capture sites were located on private rural land. Walk-in cage traps were custom built (either 1.8 x 1.8 x 0.9 or 1.2 x 0.6 x 0.5m) and baited with seed mix and apples. Each live caught bird was banded, morphometric measurements were taken and it was released at the site. Additionally, freshly dead birds (roadkill, culled) were collected in a 50 km radius around the field sites during our study: three Sulphur-crested Cockatoos, one Rainbow Lorikeet, one Galah and one Blue-winged Parrot.

Cotton bags used to hold live birds were autoclaved after each use to avoid pathogen transmission. All sampling equipment was either single-use, or thoroughly cleaned and disinfected with F10 SC Veterinary Disinfectant (Health and Hygiene (Pty) Ltd, South Africa) after each use. From each live caught individual, we took a total of about 100 μl of blood from the brachial vein. We collected part of the blood sample into 1.5 ml Screw-Cap Microcentrifuge Tubes (VWR International, U.S.A.) in ethanol and stored it at room temperature for qPCR assays. We collected another part of the blood sample with disposable micro-hematocrit capillaries (Hirschmann Laborgeräte GmbH & Co. KG, Germany) and centrifuged it for packed cell volume (PCV) analysis. We collected cloacal swabs and stored them at 4°C in the field, and transferred them into -80°C in the laboratory at the end of each field day. From dead birds, we collected pectoralis muscle and cloacal swab samples and froze them at -80°C until analysis [42].

### Sample analysis

We used an ammonium acetate DNA extraction method to extract DNA from blood, muscle and swab samples [33, 42, 49]. In short, samples were added to 250 mL of a cell lysis buffer (20mM EDTA, 120mM NaCl, 50mM TRIS-HCl, 20% SDS), with 200 units of proteinase K. After incubating for 15 h at 37°C, 4M ammonium acetate was added for protein removal, and after centrifugation (18 000g, for 15 min), ethanol was added to the supernatant for DNA precipitation. The extracted DNA was stored in low Tris-EDTA buffer (10mM Tris.HCL, 0.1 mM EDTA; pH 7.5–8.0) at -20°C [42]. We determined DNA quality and quantity using a DU 640B spectrophotometer (Beckman Coulter, CA, U.S.A), with a 1:200 dilution, and a Nanodrop 1000 spectrophotometer (Thermo Fisher Scientific, MA, U.S.A.).

We used a probe-based quantitative real-time PCR (qPCR) method for BFDV detection and quantification [42]. DNA from blood was diluted to the same concentration (200 ng/ul) to allow comparisons of viral presence and load across individuals [42]. This was not possible for swab samples due to much lower DNA yields, so we focus on reporting BFDV prevalence in swab samples. The qPCR was performed using the PikoReal Real-Time PCR System (Thermo Fisher Scientific Inc., U.S.A.). We added one positive control and one no-template control to each qPCR plate. The positive control was pooled from several confirmed BFDV positive DNA samples from previous studies at Deakin University and the Raidal lab at Charles Sturt University. The no-template control consisted of low Tris-EDTA buffer, the same type of buffer as was used to store DNA in. We ran the controls and all samples in duplicate. Duplicate samples with Cq (cycle at which probe fluorescence crosses the arbitrarily set detection baseline) differing by more than one cycle were run again. To validate that this qPCR method worked with our samples, we successfully tested known BFDV positive samples (n = 5 individuals) from three species of Australian parrots supplied by the Raidal laboratory (Charles Sturt University, Australia): one sample from a Rainbow Lorikeet, two from Sulphur-crested Cockatoos and two from Orange-bellied Parrots.

To estimate viral load, we re-ran BFDV positive blood samples on the same qPCR plate, to minimize possible variation across plates. We then used a comparative method to calculate viral load using the Cq values for each BFDV positive sample [50]. We calculated a relative estimation of viral load for each sample using the equation: Viral load = 2^(-ΔCq)^. We then log_10_-transformed the resulting viral load to achieve normality for statistical analysis, as reported in previous studies on BFDV [24].

We analyzed feathers of birds that had tested BFDV positive with qPCR with a haemagglutination (HA) assay [43, 44] to analyze excretion of BFDV antigen by infected hosts. Feathers were stored in glass vials at -20°C until transport to the Raidal laboratory at room temperature, where antigen presence and antigen titers were determined as described by Raidal et al. [43] and incorporating subsequent modifications from the Raidal lab. Feathers were placed in PBS (Phosphate-buffered saline), vortexed, then centrifuged briefly. 50 μl of the resulting supernatant was placed into the first well of each row of a 96-well microtiter plate. Each well contained 50 μl of PBS and doubling dilutions were made by transferring 50 μl of sample and PBS mix from the first well to the next well, continuing this for all wells in each row. 50 μl of 0.75% of BFDV-sensitive erythrocytes of a clinically normal Galah were added to each well. The erythrocytes had been collected into Alsever’s solution (2.05% dextrose, 0.8% sodium citrate, 0.055% citric acid, and 0.42% sodium chloride), washed in PBS, then stored in a 50:50 mix of PBS and Alsever’s. The plates were then incubated at 37°C for 45 minutes. Based on the work of Raidal et al. [43], the antigen titer was determined to be the highest dilution that caused complete haemagglutination.

### Statistical analyses

We conducted statistical analyses using SPSS 25.01 (IBM, Armonk NY, U.S.A.). We only carried out analyses with the four focal species which had the largest sample sizes. For individuals that had been trapped and sampled more than once, we only included data from the initial capture and excluded all recaptures, to ensure independence of data points. For analysis of viral load, we used all BFDV positive blood samples, which could include BFDV positive recaptures if the individual was initially BFDV negative. Each individual was thereby still only represented once for viral load. We pooled data from pectoralis muscle and blood samples for statistical analysis, as these sample types have been shown to provide comparable PCR results for BFDV [42].

In figures throughout this paper, we report the mean ± 95% confidence intervals. The statistical results in the main text are derived from generalized linear models (GLM’s) controlling for age and sex, both of which are factors known to affect BFDV prevalence [25]. To analyse effects of these predictors on prevalence, and presence of antigen excretion, we used a binomial distribution with a logit link, with ‘BFDV status’ in blood or swabs as a binary BFDV presence-absence variable. We used Gaussian models with identity link for analysis of continuous variables (viral load, PCV, antigen titers and body condition). We calculated body condition by including tarsus length, as a measure of body size, as a predictor in the model, and body mass as the dependent variable [51]. We tested body condition controlling for species and sex, as these were expected to be strong predictors of body mass in our study species [47].

## Results

**Fig 1:**
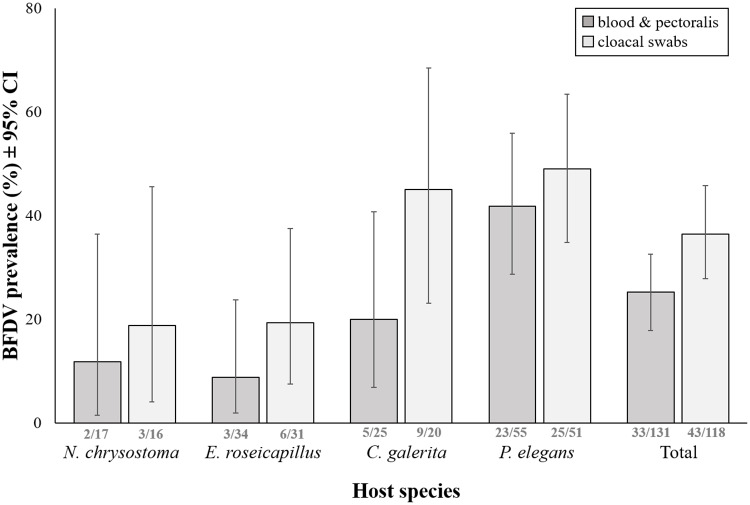
Mean BFDV prevalence (% of total individuals tested) ± 95% confidence intervals. Shown for blood and pectoralis muscle samples, as well as cloacal swabs, separately for each focal host species and pooled across the four focal host species. Data labels represent number of birds infected out of number of birds tested.

We found BFDV in six of the seven species tested (Table 1). We detected BFDV in all four focal species, namely Crimson Rosellas, Galahs, Sulphur-crested Cockatoos and Blue-winged Parrots, and prevalence in these species ranged from 8.8% to 41.8% in blood samples, and from 18.8% to 49.0% in cloacal swabs (Fig 1, Table 1). In both blood samples and cloacal swabs, BFDV prevalence differed significantly between species (blood: p = 0.006, cloacal: p = 0.023), but not between sexes (blood: p = 0.363, cloacal: p = 0.374, Table 2, S1 Fig). Pairwise comparisons between species for BFDV prevalence in both sample types showed that Crimson Rosellas had a significantly higher prevalence than Galahs (blood: p < 0.001, cloacal: p = 0.002, Fig 1, S1 Table) and Blue-winged Parrots (blood: p = 0.01, cloacal: p = 0.018, S1 Table). All other pairwise species comparisons were not significantly different. Of 55 BFDV positive birds, 21.8% (95% CI 11.8, 35.0) were BFDV positive in blood only, 49.1% (95% CI 35.4, 62.9) in cloacal swabs only, and 29.1% (95% CI 17.6, 42.9) in both sample types. Prevalence in cloacal swabs was significantly higher than in blood samples (McNemar’s χ^2^ = 5.026, df = 1, p = 0.025; Fig 1). We found BFDV in two of the three non-focal host species (Table 1), namely in Eastern Rosellas and Rainbow Lorikeets, but not in Red-rumped Parrots.

**Table 1:** BFDV prevalence combined for blood and pectoralis muscle samples, as well as in cloacal swabs, for each species tested.

species tested	no. samples	no. samples	BFDV (%)	BFDV (%)
	(blood + pectoralis)	(cloacal swabs)	in blood + pectoralis^a^	in cloacal swabs^a^
Crimson Rosella (*P*. *elegans*)	55	51	**41.8** (28.7, 55.9)	**49.0** (34.8, 63.4)
Sulphur-crested Cockatoo *(C*. *galerita*)	25	20	**20.0** (6.8, 40.7)	**45.0** (23.1, 68.5)
Galah (*E*. *roseicapillus*)	34	31	**8.8** (1.9, 23.7)	**19.4** (7.5, 37.5)
Blue-winged Parrot (*N*. *chrysostoma*)	17	16	**11.8** (1.5, 36.4)	**18.8** (4.0, 45.6)
Rainbow Lorikeet (*T*. *moluccanus*)	3	2	**66.7** (9.4, 99.2)	**0.0** (0.0, 84.2)
Eastern Rosella (*P*. *eximius*)	3	3	**0.0** (0.0, 70.8)	**66.7** (9.4, 99.2)
Red-rumped Parrot (*P*. *haematonotus*)	2	1	**0.0** (0.0, 84.2)	**0.0** (0.0, 97.5)

^a^Prevalence is shown in bold print. 95% confidence intervals (CI) are given in brackets after the prevalence. Where prevalence is 0%, one-sided 97.5% CI are given instead of 95% CI.

**Table 2:** Effects of species and sex on BFDV prevalence in blood samples and cloacal swabs. This was tested in the four focal species, and for viral load, this was tested in individuals of these four species that were BFDV positive in blood samples.

dependent variable	no. birds tested	predictor	wald χ^2^	df	p-value^a^	model fit^b^
a) BFDV in blood	128	**species**	12.439	3	**0.006**	0.17
		sex	0.826	1	0.363	
b) BFDV in cloacal swabs	116	**species**	9.569	3	**0.023**	0.128
		sex	0.789	1	0.374	
c) viral load	22	**species**	36.911	3	**< 0.001**	0.662
		sex	0.037	1	0.848	

^a^Significant results are shown in bold.

^b^For binary dependent variables, the reported model fit is the Nagelkerke R^2^ for binary variables (prevalence) and overall R^2^ created by univariate analysis of variance for continuous variables (viral load).

**Fig 2:**
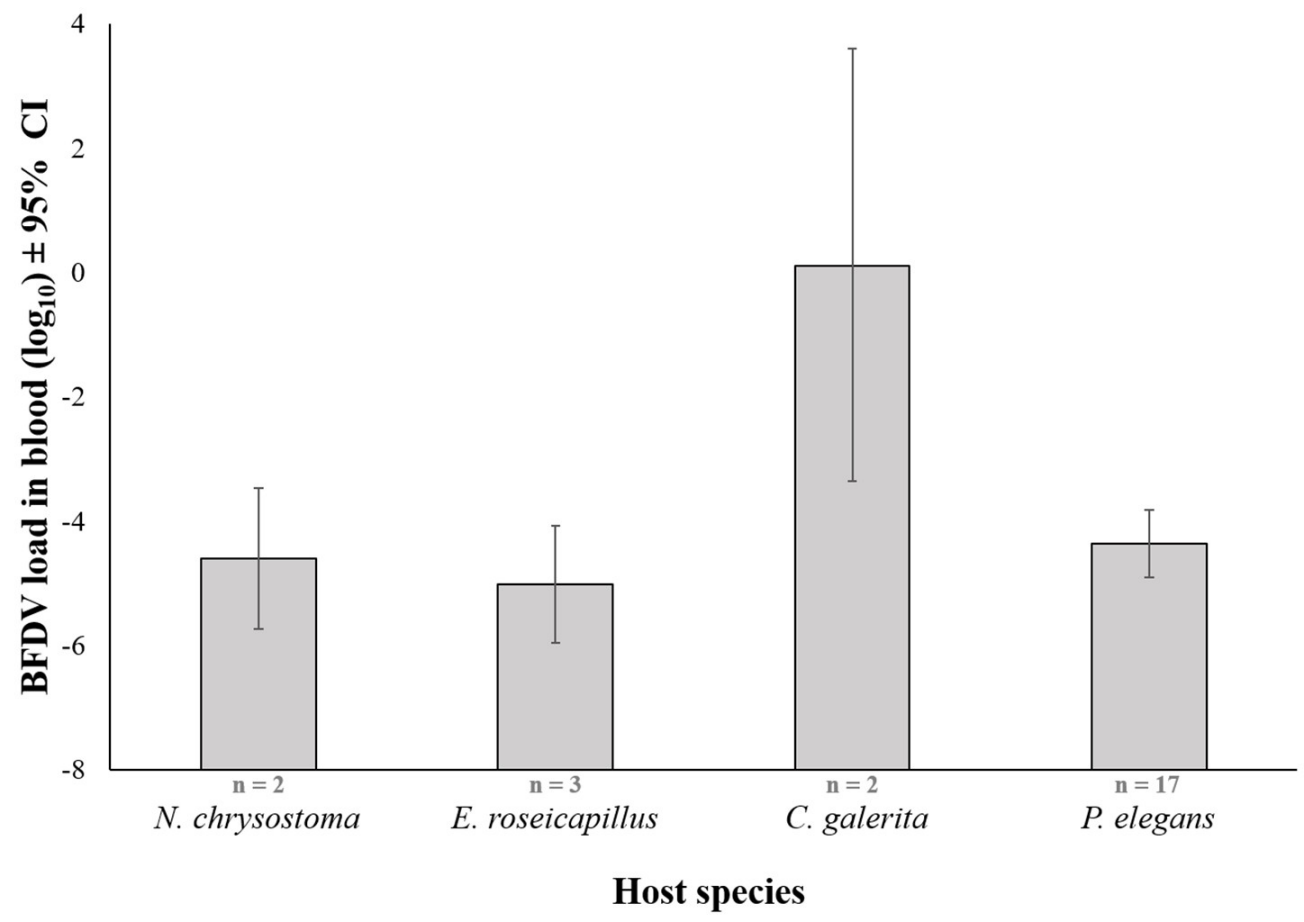
Mean viral load, shown separately for each of the four focal species. Log_10_-transformed viral load is shown ± 95% confidence intervals. Numbers at the base of bars indicate sample size of individuals for each species.

BFDV infection status (BFDV present or absent) in cloacal swabs predicted infection status in blood samples (p = 0.011), but not viral load (p = 0.426, S2 Table). Viral load showed significant interspecies differences (p < 0.001, Table 2). In the four focal species, there was no clear evidence for a correlation between viral load, and BFDV prevalence in either blood or cloacal swabs (S2 Fig). Sulphur-crested Cockatoos appeared to have the highest viral load (Fig 2), but small sample sizes for most species did not allow pairwise comparisons.

**Fig 3:**
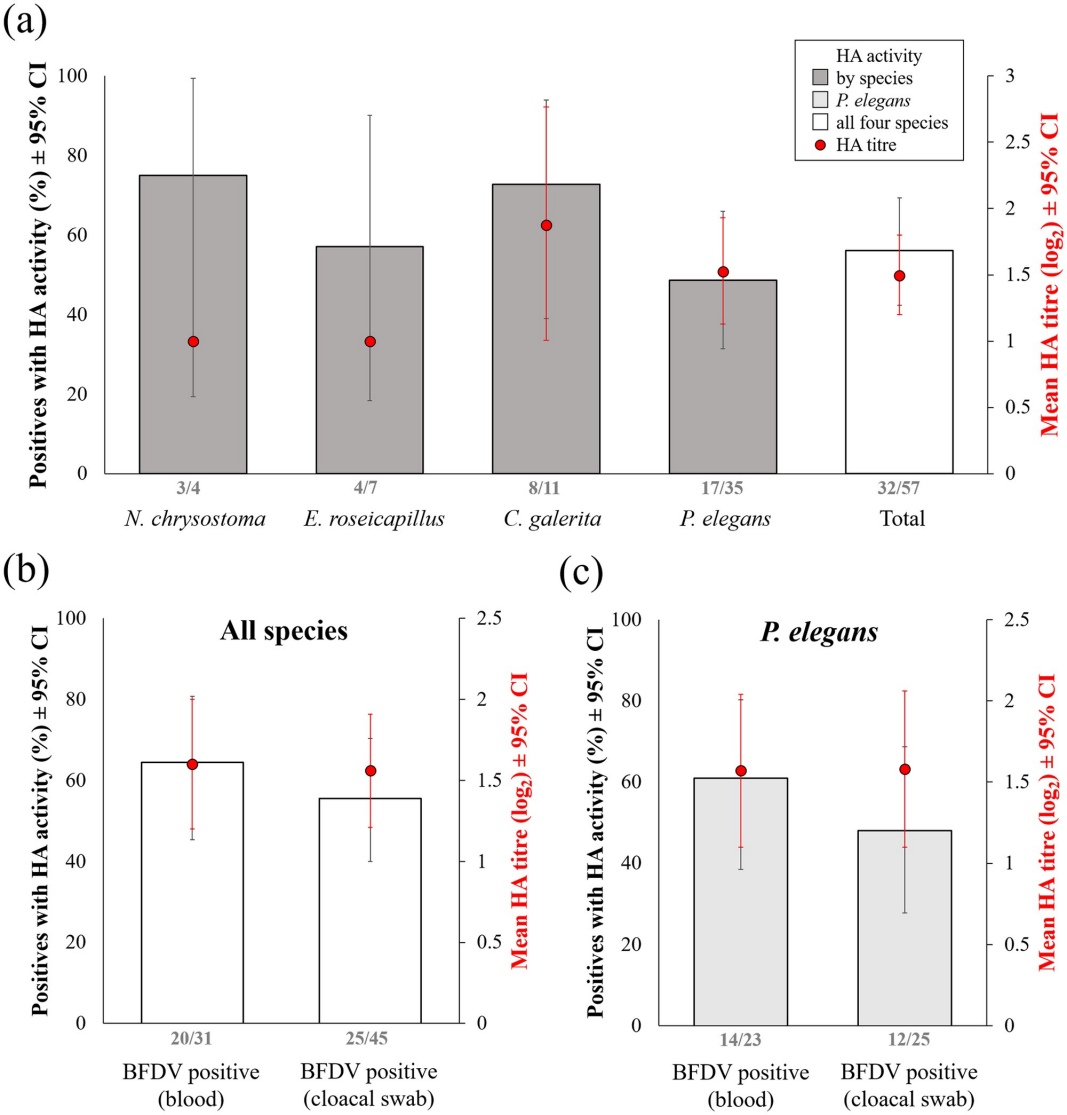
BFDV antigen excretion into feathers (positive HA result), in birds which were BFDV positive as detected by qPCR, in at least one sample type (either blood or cloacal swabs, or both). Bars show percentage of birds with antigen excretion out of all birds tested ± 95% confidence intervals, with number of birds with detectable antigen excretion out of total number of birds tested at the base of bars. Dots show average HA titre (relative amount of antigen (log_2_)) in BFDV positive birds with HA activity ± 95% confidence intervals. Percentage of birds with antigen excretion, and mean antigen titres, are shown per species (panel a), and per sample type that was BFDV positive when tested with qPCR, for all four species combined (panel b) and for Crimson Rosellas only (panel c).

Among birds of the four focal species which were BFDV positive in at least one sample type (blood, cloacal swab, or both), we found antigen excretion in feathers of 56.1% (32 of 57; 95% CI 42.4, 69.3) of individuals, with a mean antigen titer of log_2_ 1.5 (n = 32; 95% CI 1.2, 1.8) (Fig 3, S3 Table). We could only conduct statistical analyses with Crimson Rosellas, the species with the largest sample size. Crimson Rosellas that were BFDV positive in blood samples were significantly more likely to excrete antigen into feathers than those that were BFDV negative in blood samples (p = 0.032); however, there was no significant association between BFDV status of cloacal swabs and antigen excretion (p = 0.399, Fig 3, S4 Table).

There was no effect of infection status on body condition (blood: p = 0.5, cloacal: p = 0.478) or PCV (blood: p = 0.664, cloacal: p = 0.982, S5 Table). However, there were interspecies differences in PCV (p = 0.001, S3 Fig, S5 Table): Blue-winged Parrots and Galahs (trend) had a higher percentage of red blood cells than Sulphur-crested Cockatoos, and Crimson Rosellas had a lower percentage than Sulphur-crested Cockatoos, which was not significant.

## Discussion

BFDV is of major conservation concern, as it can infect most if not all members of the Psittaciformes [33], one of the most threatened bird orders world-wide [52]. Despite this, knowledge about differences in BFDV prevalence, load and excretion between host species and sexes, or the effects on host condition, is absent for the majority of natural populations [33]. This greatly hampers the understanding of the threats BFDV poses to wild birds, particularly the potential for spill-over infections from abundant to threatened species [5]. We investigated differences in BFDV prevalence and load across free living members of seven abundant parrot species in south-east Australia. We also tested whether shedding of BFDV differed between host species, if it was related to prevalence, and whether BFDV infection was associated with the sex or condition of individual hosts.

Using qPCR of blood and cloacal swabs, we detected BFDV in six of the seven host species tested. Our finding that BFDV was present in the majority of species that we tested is consistent with the results of Sutherland et al. [39], who sampled four cockatoo species caught in the wild, also in Victoria, Australia, and reported BFDV in three of the four species. Our results and those from previous studies in Australia and elsewhere indicate that BFDV has a wide host range among the Psittaciformes [18, 28]. However, most previous studies have used captive hosts or single species [28], or host individuals that were brought into veterinary care, which may be biased towards diseased birds [16]. None of these studies have tested for antigen excretion, and none have conducted statistical tests of interspecies differences in BFDV prevalence or load.

In our four focal species, prevalence was highest in Crimson Rosellas (49% in cloacal swabs and 42% in blood), followed by Sulphur-crested Cockatoos (45% and 20% respectively), Galahs (19% and 9%) and Blue-winged Parrots (19% and 9%). Other studies of wild populations of the species we tested have reported BFDV prevalence values in Sulphur-crested Cockatoos that were either much higher (92.9%, in Australia [39]) or similar (28%, in New Zealand [53]) to levels reported in our study. In New South Wales, Australia, Raidal et al. [54] reported high prevalence of antibodies against BFDV in wild populations of Sulphur-crested Cockatoos (72.4%) and Galahs (44.1%) with no evidence of clinical signs, although in other work Galahs [55] and Sulphur-crested Cockatoos [45] have been reported with severe signs of disease when infected with BFDV. In Crimson Rosellas, 34.5% prevalence was reported [42], which is similar to the 42% prevalence in blood in our study. Other studies however showed that BFDV prevalence in Crimson Rosellas is subject to substantial variation with age, subspecies [24] and breeding status [26]. To our knowledge, no studies have reported prevalence of BFDV in wild populations of Blue-winged Parrots.

BFDV has been documented in wild Australian birds for over 120 years [56]. It is thought to be endemic to Australia, having emerged there in Post-Gondwanan times [57], and having co-evolved with Australian psittacine hosts since then [5]. Interspecies differences may arise from variations in virulence in different strains of BFDV. The high mutability of BFDV has resulted in the emergence of host-specific virus strains among psittacine hosts [18, 24, 58]. Sarker et al. [22] reported a shallow host-based divergence of BFDV, and suggested that no genotype could be seen as more virulent than others. However, direct evidence relating to the potential for differences in virulence between BFDV lineages remains lacking to date. In many host-pathogen systems, degrees of resistance and tolerance vary between host species [10, 59], leading to heterogeneous prevalence and transmission rates of generalist pathogens [6]. A previous study from our group on Crimson Rosellas showed that prevalence and load can differ strongly even between subspecies, despite phylogenetic clustering of BFDV [24]. Host factors such as genotype rarity were hypothesized as the most likely mechanisms influencing these differences, rather than pathogen factors such as variation in virulence [25]. Species-specific variation in host susceptibility to BFDV is well-documented [32] and the host species used in our study are likely to have evolved some resistance or tolerance to BFDV, which could explain the often high prevalence in the absence of clinical signs, and the high prevalence of antibodies reported in other studies [54]. Alternative explanations for the differences in BFDV prevalence between studies for the same host species may arise from geographical differences in the prevalence of BFDV [9], the detection assay used [28], host age [24, 31], host behaviour [26], and the seasonal timing and location of sampling [25]. In our study, some of these potential sources of variation (e.g. sampling locations, trapping methods, detection assays) were minimized by standardisation of methods, but we could not control all factors. We could not account for host age, as in all focal species except Crimson Rosellas, the plumage colouration in youngsters and adults is similar [47], so we could not age our other study species. BFDV is often most prevalent in young birds [24, 37], but as some species cannot easily be aged, this is a limitation of studies on BFDV prevalence in these species in the wild. As the main focus of our study was to assess the distribution and prevalence in different host species, adding age data for just one species (Crimson Rosellas) would not lead to more clarity in the findings, particularly as the age effects on BFDV prevalence in load in Crimson Rosellas have been extensively studied and published elsewhere [24–26].

The use of multiple sample types and virus detection assays, as in our study, enables robust distinctions between virus carriers, which may be in a state of latent infection, and virus shedders, which may be in a state of active infection and involved in virus transmission [44]. Such distinctions are helpful for assessing the risk of transmission from infected hosts and thus the spill-over risk to co-occurring populations. Across our four focal species, we found antigen excretion in feathers in 56.1% of individuals that had tested BFDV positive with qPCR, suggesting that more than half of the viraemic birds were shedding the virus. Birds that are classified as BFDV positive when assayed only with qPCR on blood samples may not be in a state of active infection [60]. In contrast, positive haemagglutination (HA) results are considered to be an indicator for active, transmissible infection with virus replication and excretion into feather dander and feces [14], and BFDV-contaminated feather dander is one of the main sources for BFDV infection [29]. Intermittent shedding, meaning that carriers of pathogens sometimes, but not continuously, excrete high enough pathogen doses for transmission, is a common occurrence, as for example shown in *Salmonella* infections in humans [61].

Birds which are infected with BFDV but do not show signs of disease, like all birds in our study, are thought to play a major role in virus transmission, because they may shed BFDV over an extended period of time without succumbing to disease [32]. For example, wild Crimson Rosellas have been shown to remain BFDV positive in blood for at least 7.1 months as tested with qPCR, although some individuals were able to clear BFDV infection over time (data on antigen excretion were however absent [31]). We found mean antigen titers in Sulphur-crested Cockatoos (HA log_2_ 1.88), Galahs (1.0) and Crimson Rosellas (1.53) were slightly higher than antigen titers previously reported in subclinically infected Sulphur-crested Cockatoos (<1) and Galahs (<1), but much lower than in diseased birds (e.g. Sulphur-crested Cockatoos: 9.7), and such low titers may not necessarily lead to successful transmission [43]. However, when the prevalence of shedders in abundant host species is high, as found via HA testing in our study, even the excretion of low antigen titers may pose a risk to sympatric endangered species. Cloacal swabs are another commonly used method apart from HA assays to estimate viral shedding [38, 62], as BFDV and other pathogens are often transmitted via the faecal-oral route [29, 61]. BFDV prevalence was higher in cloacal swabs than in blood samples in our study, and BFDV status of cloacal swabs predicted BFDV status of blood samples. The high prevalence of BFDV positive cloacal swabs may indicate high levels of faecal shedding in addition to the shedding into feathers that is detected with the HA assay. Further testing of swabs, for example with HA assays, is needed to confirm whether BFDV shed cloacally represents viable virus, for a better understanding of shedding rates by infected hosts.

We found no sex differences in BFDV prevalence or load, in either blood samples or cloacal swabs. This is rather unexpected for several reasons. First, in many studies on wildlife disease, females are considered less susceptible to infection due to stronger immune responses [12]. The same lack of sex differences in BFDV prevalence has been shown in wild Red-fronted Parakeets (*Cyanoramphus novaezelandiae*) [27] and in a study on 19 species of captive parrots in Germany [63]. Second, studies on BFDV in wild Crimson Rosellas revealed sometimes no sex differences [25], a higher BFDV prevalence in males than in females [24], or higher BFDV prevalence in females than in males when considering breeding birds only [26]. Our findings suggest that females and males, at least of the four focal host species used in this study, and for which sample size per species was n>3, can be considered equally likely to be infected and to shed BFDV as determined by qPCR. We also found no relationships between BFDV infection and our two indices of host condition. This, together with the lack of clinical signs, suggests that BFDV infection may not have been associated with a substantial disease burden in the sample of individuals that we studied. Further research is however required, for example in additional wild populations from a wide geographical range, to confirm this.

## Conclusions

Our study provides novel information about BFDV prevalence, load and shedding in free living populations. We show that BFDV has a wide host range and is highly prevalent in wild populations of abundant psittacine species in south-east Australia, but that prevalence and shedding of BFDV differ between sympatric host species. These results suggest there may be differences in susceptibility to BFDV, or force of infection, between these host species. Overall, our findings provide new insights into the dynamics of a host generalist avian virus causing global conservation concern, and should benefit disease management particularly where there is a risk of BFDV spill-over from abundant host species to threatened populations.

## Supporting information

S1 Table Pairwise comparisons by species of BFDV prevalence in blood (no. of tested individuals = 128) and in cloacal swabs (no. of tested individuals = 116). (DOCX)

S2 Table Association between BFDV detection in blood samples and cloacal swabs, and between viral load in BFDV positive blood samples and BFDV detection in cloacal swabs. (DOCX)

S3 Table Percentage of birds with antigen excretion, and amount (titre) of antigen excretion in birds with BFDV positive (BFDV+) blood samples and cloacal swabs, measured with haemagglutination assay on feathers. (DOCX)

S4 Table For Crimson Rosellas, factors predicting BFDV antigen presence (percentage of birds with antigen excretion) and antigen titer in feather samples of BFDV positive Crimson Rosellas. (DOCX)

S5 Table Effect of infection status by sample type, as well as species and sex, on body condition and PCV. (DOCX)

S1 Fig Mean BFDV prevalence with 95% confidence intervals, by species and sex, in a) blood samples and b) cloacal swabs. Numbers at the base of bars are number of BFDV positive birds out of total number of birds tested.
(TIF)

S2 Fig Association between mean BFDV load and mean prevalence in blood (panel a, r(3) = 0.066, p (two-tailed) = 0.934) and cloacal swabs (panel b, r(3) = 0.564, p (two-tailed) = 0.436). (TIF)

S3 Fig Mean PCV by species and infection status, with 95% confidence intervals. Dark grey bars indicate birds that were BFDV positive in at least one sample type (blood, cloacal swab, or both), light grey bars indicate BFDV negative birds. PCV over 50% indicates a higher percentage of red blood cells than serum. Numbers at the base of bars indicate sample size.
(TIF)
