# Supplementary material for: Beak and feather disease virus (BFDV) prevalence, load and excretion in seven species of wild caught common Australian parrots
Source: PLoS One. 2020 Jul 1;15(7):e0235406. doi: 10.1371/journal.pone.0235406 (PMC7329075; doi:10.1371/journal.pone.0235406)
Supplement: S1 Table — (DOCX) [file pone.0235406.s001.docx]

**S1 Table. Pairwise comparisons by species of BFDV prevalence in blood (no. of tested individuals = 128) and in cloacal swabs (no. of tested individuals = 116).**

| **Sample type** | **Species^a^** | **Vs. species** | **Mean Δ^b^** | **SE** | **df** | ***p*** | **95% Wald CI for Δ** |
| --- | --- | --- | --- | --- | --- | --- | --- |
| Blood | PE | NC | -0.29 | 0.113 | 1 | **0.01** | -0.51, -0.07 |
|  | PE | ER | -0.34 | 0.084 | 1 | **< 0.001** | -0.50, -0.18 |
|  | PE | CG | -0.2 | 0.111 | 1 | 0.065 | -0.42, 0.01 |
|  | NC | ER | -0.05 | 0.104 | 1 | 0.627 | -0.25, 0.15 |
|  | NC | CG | 0.08 | 0.126 | 1 | 0.502 | -0.16, 0.33 |
|  | ER | CG | 0.14 | 0.101 | 1 | 0.179 | -0.06, 0.33 |
| Cloacal swabs | PE | NC | -0.3 | 0.126 | 1 | **0.018** | -0.54, -0.05 |
|  | PE | ER | -0.31 | 0.101 | 1 | **0.002** | -0.51, -0.11 |
|  | PE | CG | -0.05 | 0.137 | 1 | 0.728 | -0.32, 0.22 |
|  | NC | ER | -0.01 | 0.128 | 1 | 0.925 | -0.26, 0.24 |
|  | NC | CG | 0.25 | 0.158 | 1 | 0.114 | -0.06, 0.56 |
|  | ER | CG | 0.26 | 0.138 | 1 | 0.059 | -0.01, 0.53 |

^a^Species abbreviations are as follows*: P. elegans* (PE), *N. chrysostoma* (NC), *E. roseicapillus* (ER), *C. galerita* (CG).

^b^We used Δ as the symbol for difference.
