# Supplementary material for: Beak and feather disease virus (BFDV) prevalence, load and excretion in seven species of wild caught common Australian parrots
Source: PLoS One. 2020 Jul 1;15(7):e0235406. doi: 10.1371/journal.pone.0235406 (PMC7329075; doi:10.1371/journal.pone.0235406)
Supplement: S2 Table — (DOCX) [file pone.0235406.s002.docx]

**S2** **Table. Association between BFDV detection in blood samples and cloacal swabs, and between viral load in BFDV positive blood samples and BFDV detection in cloacal swabs.**

| **Dependent variable** | **No. birds tested** | **Predictor** | **Wald χ^2^** | **df** | ***p*** | **Model fit^a^** |
| --- | --- | --- | --- | --- | --- | --- |
| BFDV status (blood) | 118 | BFDV status (cloacal swab) | 6.482 | 1 | **0.011** | 0.082 |
| Viral load | 22 | BFDV status (cloacal swab) | 0.632 | 1 | 0.426 | 0.028 |

^a^For binary dependent variables, the reported Model fit is the Nagelkerke R^2^, for viral load it is the partial Eta^2^.
