# Supplementary material for: Beak and feather disease virus (BFDV) prevalence, load and excretion in seven species of wild caught common Australian parrots
Source: PLoS One. 2020 Jul 1;15(7):e0235406. doi: 10.1371/journal.pone.0235406 (PMC7329075; doi:10.1371/journal.pone.0235406)
Supplement: S3 Table — (DOCX) [file pone.0235406.s003.docx]

**S3 Table. Percentage of birds with antigen excretion, and amount (titer) of antigen excretion in birds with BFDV positive (BFDV+) blood samples and cloacal swabs, measured with Haemagglutination (HA) assay on feathers.**

| **Subset tested^a^** | **No. birds**  **tested^b^** | **No. HA+**  **birds^b^** | **HA+ birds (%)** | **HA+ (%)**  **95% CI** | **Mean**  **HA titre** | **HA titre**  **95% CI** |
| --- | --- | --- | --- | --- | --- | --- |
| **Species** |  |  |  |  |  |  |
| *N. chrysostoma* | 4 | 3 | 75.0 | 19.4, 99.4 | 1.00 | 1.00, 1.00 |
| *E. roseicapillus* | 7 | 4 | 57.1 | 18.4, 90.1 | 1.00 | 1.00, 1.00 |
| *C. galerita* | 11 | 8 | 72.7 | 39.0, 94.0 | 1.88 | 1.00, 2.76 |
| *P. elegans* | 35 | 17 | 48.6 | 31.4, 66.0 | 1.53 | 1.13, 1.93 |
| all four species | 57 | 32 | 56.1 | 42.4, 69.3 | 1.50 | 1.20, 1.80 |
| **All four species combined, by BFDV+ sample type** | | | | | | |
| blood | 30 | 19 | 63.3 | 43.9, 80.1 | 1.53 | 1.14, 1.92 |
| cloacal swabs | 43 | 25 | 58.1 | 42.1, 73.0 | 1.56 | 1.20, 1.92 |
| ***P. elegans*, by BFDV+ sample type** | | | | | | |
| blood | 23 | 14 | 60.9 | 38.5, 80.3 | 1.57 | 1.10, 2.04 |
| cloacal swabs | 25 | 12 | 48.0 | 27.8, 68.7 | 1.58 | 1.10, 2.06 |

^a^We show HA data by species, and by sample type that was BFDV positive when tested with a qPCR assay.

^b^Abbreviations are as follows: Number (no.), positive haemagglutination result (HA+).
