# Supplementary material for: Beak and feather disease virus (BFDV) prevalence, load and excretion in seven species of wild caught common Australian parrots
Source: PLoS One. 2020 Jul 1;15(7):e0235406. doi: 10.1371/journal.pone.0235406 (PMC7329075; doi:10.1371/journal.pone.0235406)
Supplement: S4 Table — (DOCX) [file pone.0235406.s004.docx]

**Table S4. For Crimson Rosellas, factors predicting presence (percentage of birds with antigen excretion) and amount (titer) of BFDV antigen in feather samples of BFDV positive Crimson Rosellas.**

| **Dependent variable** | **No. birds tested** | **Predictor** | **Wald χ^2^** | **df** | ***p*** | **Model fit^b^** |
| --- | --- | --- | --- | --- | --- | --- |
| Antigen presence | 35 | BFDV status (blood)^a^ | 4.616 | 1 | **0.032** | 0.194 |
|  |  | BFDV status (cloacal swab) | 0.71 | 1 | 0.399 |  |
|  |  | sex | 0.4 | 1 | 0.527 |  |
| Antigen titer | 17 | BFDV status (blood) | 0.7 | 1 | 0.403 | 0.068 |
|  |  | BFDV status (cloacal swab) | 0.643 | 1 | 0.422 |  |
|  |  | sex | 0.592 | 1 | 0.442 |  |

^a^’BFDV status’ is a categorical predictor showing in which sample type individuals were BFDV positive.

^b^For binary dependent variables, the reported Model fit is the Nagelkerke R^2^ for antigen presence, for antigen titre it is the overall R^2^ calculated by univariate analysis of variance.
