# Supplementary material for: Beak and feather disease virus (BFDV) prevalence, load and excretion in seven species of wild caught common Australian parrots
Source: PLoS One. 2020 Jul 1;15(7):e0235406. doi: 10.1371/journal.pone.0235406 (PMC7329075; doi:10.1371/journal.pone.0235406)
Supplement: S5 Table — (DOCX) [file pone.0235406.s005.docx]

**Table S5.** Effect of infection status by sample type, as well as species and sex, on body condition and packed cell volume (PCV).

| **Dependent variable** | **No. birds tested** | **Predictor** | **Wald χ^2^** | **df** | ***p*** | **Model fit^b^** |
| --- | --- | --- | --- | --- | --- | --- |
| Body condition | 60 | BFDV status (blood)^a^ | 0.454 | 1 | 0.5 | 0.997 |
|  |  | BFDV status (cloacal swab) | 0.504 | 1 | 0.478 |  |
|  |  | species | 5400.457 | 3 | **< 0.001** |  |
|  |  | sex | 18.128 | 1 | **< 0.001** |  |
|  |  | Tarsus length | 34.644 | 1 | **< 0.001** |  |
| PCV | 100 | BFDV status (blood) | 0.189 | 1 | 0.664 | 0.166 |
|  |  | BFDV status (cloacal swab) | 0.001 | 1 | 0.982 |  |
|  |  | species | 16.846 | 3 | **0.001** |  |
|  |  | sex | 0.099 | 1 | 0.753 |  |

^a^’BFDV status’ is a categorical predictor showing in which sample type individuals were BFDV positive.

^b^The reported model fit is the overall R^2^ calculated by univariate analysis of variance.
