# Supplementary figures and images for: Beak and feather disease virus (BFDV) prevalence, load and excretion in seven species of wild caught common Australian parrots
Source: PLoS One. 2020 Jul 1;15(7):e0235406. doi: 10.1371/journal.pone.0235406 (PMC7329075; doi:10.1371/journal.pone.0235406)

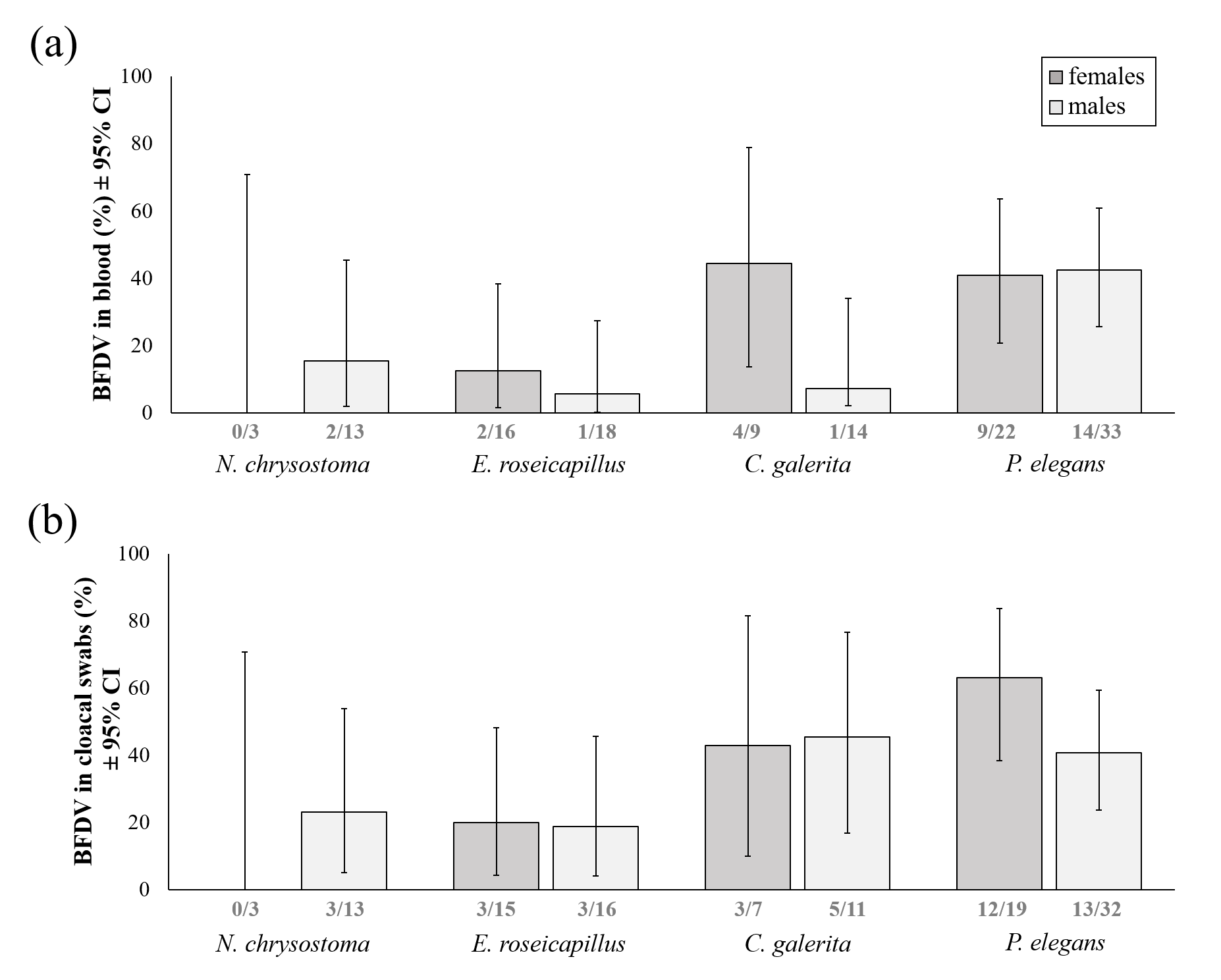

Supplement: S1 Fig — Numbers at the base of bars are number of BFDV positive birds out of total number of birds tested. (TIF) [file pone.0235406.s006.tif]

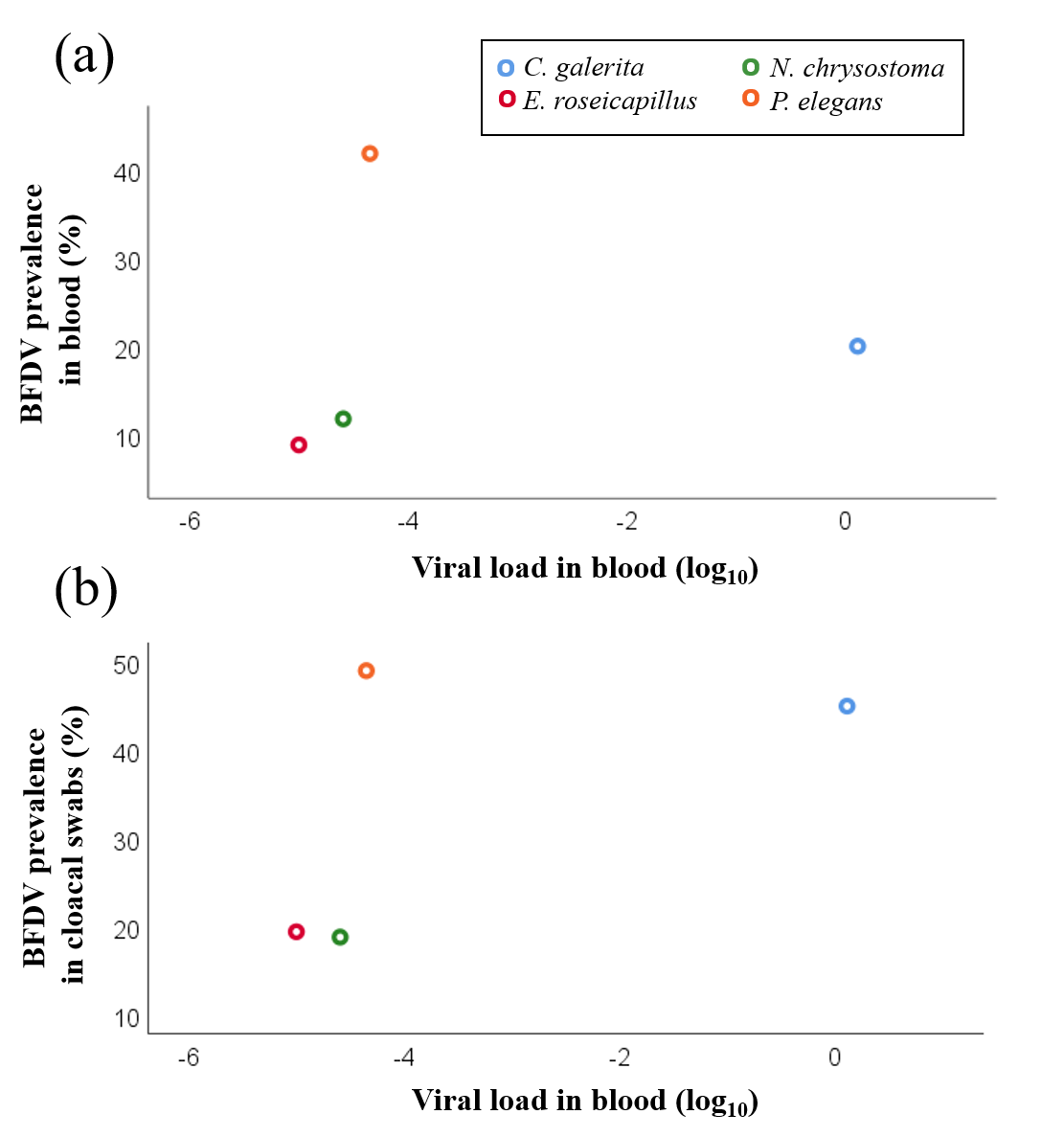

Supplement: S2 Fig — (TIF) [file pone.0235406.s007.tif]

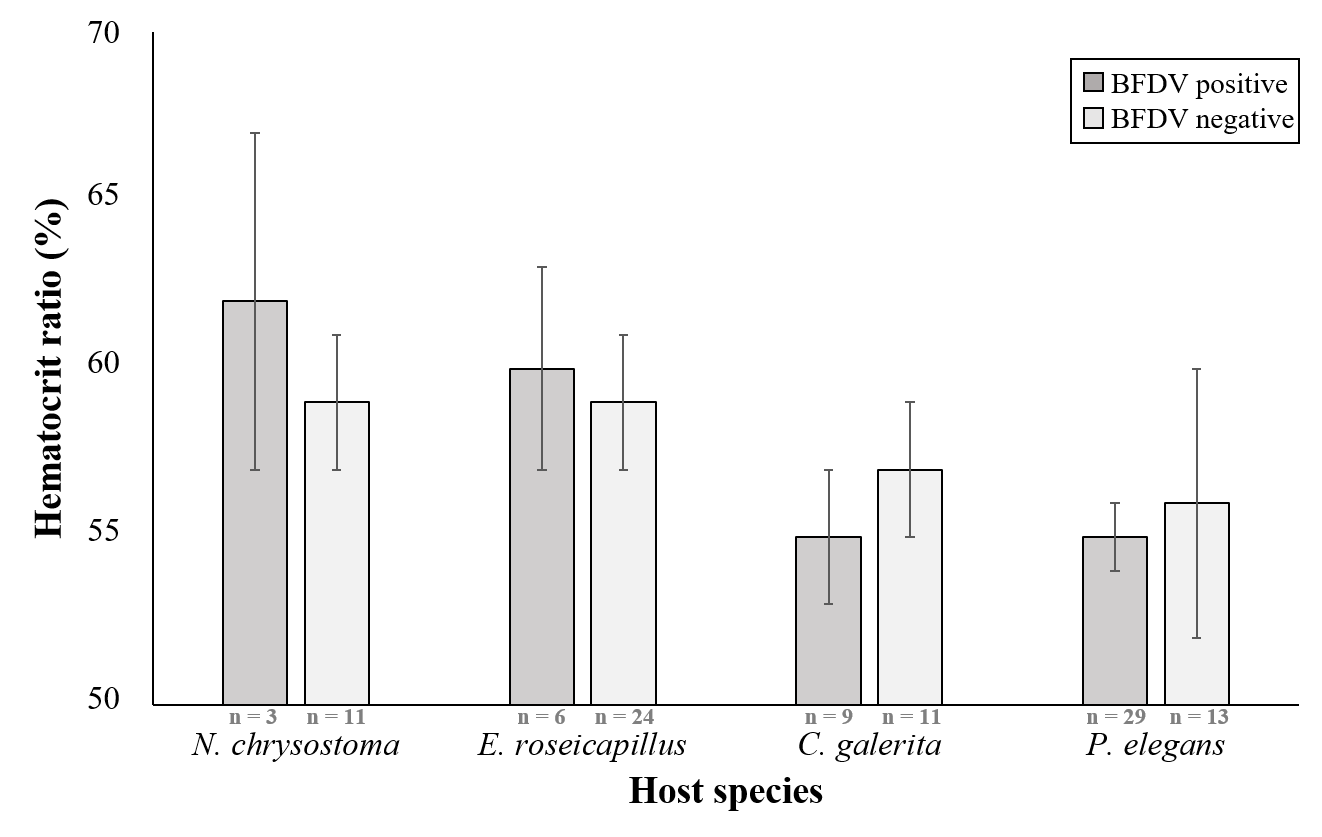

Supplement: S3 Fig — Dark grey bars indicate birds that were BFDV positive in at least one sample type (blood, cloacal swab, or both), light grey bars indicate BFDV negative birds. PCV over 50% indicates a higher percentage of red blood cells than serum. Numbers at the base of bars indicate sample size. (TIF) [file pone.0235406.s008.tif]
